# Dynamic Length Changes of Telomeres and Their Nuclear Organization in Chronic Myeloid Leukemia

**DOI:** 10.3390/cancers5031086

**Published:** 2013-08-22

**Authors:** Oumar Samassekou

**Affiliations:** Manitoba Institute of Cell Biology, Cancer Care Manitoba, Department of Physiology, University of Manitoba, Winnipeg, Manitoba R3E 0V9, Canada; E-Mail: samasseo@cc.umanitoba.ca; Tel.: +1-204-787-4125; Fax: +1-204-787-2190

**Keywords:** chronic myeloid leukemia, telomere lengths, telomerase, ALT, telomeric nuclear organization

## Abstract

Chronic myeloid leukemia (CML) is a myeloproliferative neoplasm characterized by the t(9;22) translocation. As in most cancers, short telomeres are one of the features of CML cells, and telomere shortening accentuates as the disease progresses from the chronic phase to the blastic phase. Although most individual telomeres are short, some of them are lengthened, and long individual telomeres occur non-randomly and might be associated with clonal selection. Telomerase is the main mechanism used to maintain telomere lengths, and its activity increases when CML evolves toward advanced stages. ALT might be another mechanism employed by CML cells to sustain the homeostasis of their telomere lengths and this mechanism seems predominant at the early stage of leukemogenesis. Also, telomerase and ALT might jointly act to maintain telomere lengths at the chronic phase, and as CML progresses, telomerase becomes the major mechanism. Finally, CML cells display an altered nuclear organization of their telomeres which is characterized by the presence of high number of telomeric aggregates, a feature of genomic instability, and differential positioning of telomeres. CML represents a good model to study mechanisms responsible for dynamic changes of individual telomere lengths and the remodeling of telomeric nuclear organization throughout cancer progression.

## 1. Telomere Overview

The ends of human chromosomes, called telomeres, are constituted of a tandem of repeats TTAGGG and nucleoprotein complexes, and they play a crucial role in cellular homeostasis by maintaining genome stability and integrity. These telomeric functions cannot be attained unless telomere lengths are maintained at a level that allows telomeres to avoid chromosome end-to-end fusion, DNA cascade signaling, and genomic instability [1].

In healthy individuals, the average length of telomeres ranges between 5 and 15 kb, and can differ among individuals, tissues, cells, chromosomes, and chromosome arms. Average length of telomeres (telomeres from groups of cells, tissues, or organs) and length of individual telomere (telomeres on each chromosome arm) can be assessed by using genomic DNA or cytological preparations [2]. Both *in vitro* and *in vivo* studies have shown negative correlation between telomere length and cellular aging [3,4,5]. In normal stem cells and germ lines, telomeres are maintained by a special ribonucleoprotein enzyme, called telomerase, which counteracts loss of telomeric sequences by adding telomere repeats at the 3' telomeric overhang [6,7]. Telomerase is composed of a catalytic unit, (TERT), and an RNA unit, TERC, which serves as a substrate for telomere elongation. In absence of telomerase, telomere shortening can lead to the disruption of telomere structure, telomere fusions, and a cascade of DNA damage signaling. Then, cells enter into senescence, apoptosis, or crisis [8,9,10]. Cells can bypass crisis by activating mechanisms of telomere length maintenance, telomerase or alternative lengthening of telomeres (ALT), and further genomic instability can induce cell transformation and tumor initiation [2].

In most cancers, telomerase is activated and maintains telomere length homeostasis to ensure cell proliferation [11,12]. ALT is the other telomere length maintenance mechanism, present in 15% of tumors [13,14]. ALT cells present at least one of these phenotypes: heterogeneous telomere lengths [15], telomeric DNA and shelterin proteins associated with promyelocytic leukemia bodies (APB) [16,17], numerous double-stranded and C-rich telomeric circles [18,19,20], increased numbers of DNA damage response foci at telomeres [21], and an increased frequency of telomeric sister-chromatid exchanges [22,23].

Although mechanisms of telomere length maintenance are always activated in tumors, the presence of short telomeres is one of the hallmarks of neoplastic cells and they can induce or exacerbate genomic instability [24,25,26,27]. Likewise telomere shortening, the alteration of telomeric nuclear organization has been associated with genomic instability and cancer progression [28,29,30,31,32,33]. Telomeric nuclear organization is defined by: (1) the number of telomeres (telomere signals), (2) telomere length (telomere signal intensity), (3) the number of telomere aggregates (TAs) (telomere clusters, found in close proximity that cannot be further resolved as separate entities at an optical resolution limit of 200 nm), (4) telomere distribution within a nucleus, and (5) telomere positions (the distance of each telomere from the nuclear center versus the periphery) [28,32,34,35].

Chronic myeloid leukemia (CML) is one of the rare cancers whose telomere biology (average length of telomeres, length of individual telomeres, different mechanisms of telomere length maintenance, and telomeric nuclear organization) has been extensively studied. Integrating knowledge covering different aspects of telomere biology in CML would allow one to gain deeper insights into how intertwined different components of telomere biology are in cancer, generally, and CML, particularly. In this review, we will discuss changes in telomere lengths, their maintenance mechanisms, and telomeric nuclear organization in CML. This pathology offers a unique model to study different facets of telomere biology in cancer because of its well characterized natural history, the easy access of tumor cells, the availability of control tissue (same cellular origin), the good quality of karyotype (necessary for the measurement of individual telomere lengths), and the availability of good therapeutic which can enable to study the impact of successful treatment on telomere biology.

## 2. Clinical Presentation and Molecular Biology of Chronic Myeloid Leukemia

Chronic myeloid leukemia is a myeloproliferative neoplasm and characterized by an excessive proliferation of myeloid cells in bone marrow and their accumulation in blood. Its incidence is 1 to 1.5 per 100,000 people, and it mainly affects adults between 50 and 60 years old. The cause of CML is unknown, but exposures to ionizing radiation and benzene have been reported as risk factors [36,37]. Clinically, patients present with generalized fatigue, weight loss, night sweats, anemia, splenomegaly, and unexplained bleeding. More than half of the patients are asymptomatic at their diagnosis, and over 90% of the patients are diagnosed at the chronic phase (CP). If no appropriate treatment is administered, CML evolves irremediably in three clinical phases: the chronic phase (CP), the accelerated phase (AP), and the blastic phase (BP) [38]. The current treatment of CML is primarily based on molecular targeted therapies, specifically tyrosine kinase inhibitors, and this therapeutic approach has the advantage of being more effective than bone marrow transplantation and chemotherapy, as well as being less toxic than the latter [39].

CML is a clonal disease and characterized by the presence of the Philadelphia chromosome (Ph), resulting from the reciprocal translocation t(9;22)(q34.1;q11.2) [40]. This translocation is responsible for the fusion of the oncogene c-abl-1 non-receptor tyrosine kinase (*ABL1*) located on chromosome 9 with the breakpoint cluster region (*BCR*) on chromosome 22 [41]. In most patients, the fusion gene *BCR-ABL1* produces a 210 kDa protein which has a highly constitutive tyrosine kinase activity. This high tyrosine kinase activity leads to activation of mitogenic signaling pathways, altered cell adhesion, inhibition of apoptosis, and arrest of cell differentiation [42]. When no appropriate treatment is administered, CML irreversibly progresses from CP to the AP and to the BP. These two latter phases are characterized by the appearance of secondary chromosomal abnormalities: +8, +Ph, i(17q), +19, −Y, +21, +17, and −7 [43,44].

The molecular drivers of CML evolution are poorly understood, and some authors have suggested an evolution in a stepwise fashion. An increased activity of the fusion protein BCR-ABL1 might be followed by inactivation of tumor suppressor genes such as *TP53*, a default in DNA repair machinery, an emergence of other chromosomal abnormalities, and occurrence of genomic instability [38]. Data from gene expression profiling have indexed six key genes (*NOB1*, *DDX47*, *IGSF2*, *LTB4R*, *SCARB1*, and *SLC25A3*) which have been suggested to discriminate the initial and late stages of each clinical phase of CML [45], but their role in the progression of CML remains unknown. Nevertheless, knowledge gained from the molecular pathogenesis of CML has enabled the development of the first molecular target therapy, imatinib mesylate (Gleevec^®^, Novartis Pharmaceuticals Corporation, NJ, USA), and subsequently, other similar molecules have been developed against emerging resistant clones [39].

## 3. Telomere Length in Chronic Myeloid Leukemia

### 3.1. Average Length of Telomeres

In CML, most of the studies have assessed the average length of telomeres by using either telomere restriction fragment (TRF) or fluorescence *in situ* hybridization (FISH) coupled with flow cytometry (Flow-FISH) (Table 1). The genomic DNA is used for TRF, and the technique has the advantage to estimate the physical length of telomeres in kb, enabling comparison of telomere lengths between different studies. For the flow-FISH, cytological preparations are used, and it has the benefit of comparing telomere lengths of different cellular populations within the same preparation [2].

**Table 1:** Comparison of widely used techniques for the measurement of telomere lengths in CML.

	Techniques	Materials	Advantages	Disadvantages
**Measurements of average telomeres**	TRF	DNA	Comparison between studies	Large amount of DNA Labor intense
	Flow FISH	Interphase cells	Can measure different cell subsets	Require cytological preparation
**Measurements of individual telomeres**	Q-FISH	Metaphases	Can identify all individual telomeres	Requires metaphases
	3D telomere FISH	Interphase cells	Assess the number, intensity and the position of individual telomeres in interphase nuclei	Cannot identify individual telomeres

Many studies have found that telomeres of leukemic cells from CML patients are shorter than those of white blood cells from healthy individuals [46,47,48,49]. Among these studies, one reported that telomere length difference between leukemic cells from CML patients and leucocytes from aged-matched healthy individuals can reach one kb. The same study compared telomere lengths of Ph positive cells and Ph negative T lymphocytes from the same CML patient, and showed telomeres of leukemic cells are shorter than those of Ph negative T lymphocytes [47]. Furthermore, average telomere length has been reported to negatively correlate with disease progression. Indeed, patients in PA and PB presented significantly shorter telomeres than those in the PC [47,50,51]. Finally, short telomeres have been associated with poor prognostic and high score of Hasford [49].

Many factors have been proposed to account for telomere shortening in CML. Like other cancers high proliferation rate of leukemic cells has been suggested of being the prominent driving force for telomere shortening [51]. Moreover, BCR-ABL1 might influence telomere shortening during different phases of CML. Increased activity of tyrosine kinase can generate reactive oxygen species which could lead to oxidative damage and telomere shortening [52]. Finally, the uncapping of telomeres through disruption of shelterin proteins which are altered during CML evolution [53] might be another cause for telomere shortening.

A successful treatment of CML with tyrosine kinase inhibitors has been associated with telomere lengthening due to the decline of Ph positive cells in blood and bone marrow [48]. Although telomeres of CML patients lengthen after successful treatment, telomeres of the myeloid compartment still shorter than their counterparts in healthy individuals. A recent study showed that telomeres of myeloid cells (Ph negative) from CML patients in remission, after successful treatment, were shorter than those of age-matched healthy controls whereas telomere lengths of lymphoid cells did not present any statistical difference between the patients and the controls [54]. The authors explained the persistence of short telomeres in the myeloid compartment by diverse factors not being exclusive: (1) presence of intrinsic short telomeres of myeloid stem cells before transformation; (2) effect of myeloid microenvironment; and (3) accrued proliferation of myeloid stem cells [54]. It would be interesting to know if the persistence of short telomeres in myeloid compartment can have deleterious consequences on patients in remission.

### 3.2. Length of Individual Telomeres

Few studies have been done in CML to determine lengths of individual telomeres, and this scarcity of data regarding individual telomere lengths is also common in others cancers. Two recent studies have provided new insights into the profiling of individual telomere lengths in CML [46,55]. Both of these studies found different profiles between healthy individuals and CML patients at the CP. In CML, individual telomeres on 18p and Xp were the longest while the shortest were on 20q, 21p and 21q [46]. On the other hand, in healthy individuals, telomeres on 17p, 19p, and 20q were the shortest while those on 5p, 3p, 4q, and 1p were the longest [46,56,57]. These results suggest that dynamic of telomere shortening or lengthening is different between normal and leukemic cells.

Individual telomeres might present different shortening rates during leukomogenesis. By using statistical modeling and measurements of individual telomere lengths from CML patients at the CP and healthy aged-matched controls, a study estimated that individual telomeres present different shortening rates from CML initiation to the diagnostic (CP) [46]. For instance, telomeres on Yp, Yq, 1q, 5q, 9q, 8p, and 21p presented the highest telomere attrition rates, and their lengths were at least 30% shorter than their counterparts in healthy population. Then, it was hypothesized that the pronounced shortening of telomeres on both arms of the Y chromosome, which is one of the recurrent secondary abnormalities in CML [43,58] could explain its loss at the late stages of CML [46]. Also, telomere shortening on some specific chromosome arms might account for some secondary chromosomal abnormalities during CML progression [46].

Most notably, studies on individual telomere lengths have highlighted the presence of long telomeres on some specific chromosomes arms, features of some CML samples. For instance, telomeres on Xp, 5p, 7q and 3p recurrently lengthened in some samples, and their length ranged from 14.1 Kb to 24.8 Kb while the average lengths of telomeres in those samples were between 6–8 Kb [46,55]. Also, these long telomeres had the particularities to be mono allelic and were present in a proportion of cells, evoking clonal selection [55]. These long individual telomeres in CML at the CP might be a consequence of clonal expansion and associated with CML progression from CP to BP. A similar observation was made in a case of B type acute lymphoblastic leukemia which displayed a very long telomere on one of the 11q, and this long telomere, subsequent to the t(9;22) translocation, was associated with disease progression and clonal expansion [59]. On the other hand, selective lengthening of individual telomeres may be a cause of clonal selection in CML. The lengthening of telomeres on some chromosome arms may lead to telomere position effects [60,61] and down regulate the expression of certain genes at subtelomeric regions. For instance, some of these genes might carry out antiproliferative functions, so their down regulation might confer a proliferative advantage to cells harboring these long individual telomeres.

The recurrent shortening or lengthening of individual telomeres may serve as a marker for clinical monitoring in CML and other cancers. Further studies evaluating their clinical values in different clinical stages of CML and other tumors are needed before establishing their usefulness in the clinical setting. Moreover, it would be essential for cancer studies to better elucidate different mechanisms underlying telomere shortening or lengthening at chromosomal-arm level. Also, the understanding of eventual roles of individual telomere shortening or lengthening in chromosomal abnormalities, clonal expansion, and cell proliferation would expand our knowledge on genomic instability and cell survival in cancer.

## 4. Mechanisms of Telomere Maintenance

### 4.1. Telomerase

In CML, telomerase had been proposed to be the only telomerase maintenance mechanism before a recent study suggested that ALT may also play a role in maintaining telomere lengths [62]. The telomerase is activated in CP patients and its activity increases as the disease progresses from the CP to the AP and from the AP to the BP. This high telomerase activity is probably due to an increased number of blasts and has been associated with poor prognosis [63,64,65]. Furthermore, genomic instability might be another reason for poor prognostic in patients presenting high telomerase activity. In fact, an elevation of telomerase activity has been associated with the acquisition of new cytogenetic abnormalities [64], and more than 60% of CML patients with high telomerase activity presented microsatellite instability [66]. Thus, a surge in telomerase activity may be associated with genomic instability and aggravate the neoplastic process during CML evolution.

Tyrosine kinase inhibitors, specifically those targeting BCR-ABL1, might be involved in telomerase regulation. Treatment of different cell lines expressing telomerase by imatinib down regulates telomerase activity and inhibits cell proliferation suggesting direct action of imatinib on telomerase regulation [67]. Moreover, *in vitro* treatment of leukemic cells from CML patients by imatinib represses telomerase activity more in cells from CP patients than in those from BP patients. The same study showed that inhibition of telomerase was due to a direct action of imatinib on *TERT* transcription [68]. Another study reported imatinib induces a transcriptional repression of *TERT* only in imatinib-sensitive cells but not in imatinib-resistant cells, and telomerase was suggested to represent an additional factor for imatinib resistance in blast crisis. This resistance occurs faster when cells overexpress *TERT* [69]. Finally, BCR-ABL was proposed to regulate telomerase activity, and this regulation occurs at multiple levels, including transcription, at the post-translational level, and proper localization [70]. In conclusion, BCR-ABL1 might induce or enhance telomerase activity leading to sustained cell proliferation and cell resistance to apoptosis.

An association of anti-telomerase therapy with standard treatment in CML could assure better clinical outcome by overcoming therapeutic resistance of anti-tyrosine kinase. An *in vitro* study has shown that inhibition of the catalytic unit of telomerase (TERT) in leukemic cells potentiates the cis-diamminedichloroplatinum effect by increasing apoptosis [71]. Divers strategies such as transcriptional inhibition of essential telomerase components by short interfering RNA (siRNA), inhibition of telomerase activity by dominant negative of TERT or small molecules, and use of antisense-oligodesoxynucleotides against the RNA component of telomerase (TERC) have been proposed for telomerase inhibition in CML [51]. However, leukemic cells which might use mechanisms other than telomerase to maintain their telomeres [62] could impede clinical success of anti telomerase therapy in CML. Integrative strategies should be developed to better target anti-telomere maintenance mechanisms.

### 4.2. Alternative Lengthening of Telomere

Rarely has the presence of the ALT mechanism been reported in hematological malignancies, and only one study has investigated this mechanism in CML. The authors of this study had based their hypothesis for ALT involvement in CML on the fluctuation of length ratios of intra-arm or inter-homologous telomeres, found in their previous studies [46,55]. Then, they studied the presence of ALT in CML patients at the CP by identifying telomeric C-circles, the most specific ALT marker [14,72]. In 3 out 24 samples, C-circles were present in absence of telomerase activity, and ALT was suggested as the sole mechanism maintaining telomere length in these samples. Moreover, 6 out 24 samples presented double strand telomeric circles when telomerase is activated or not [62]. These double strand telomeric circles might either be suggestive of ALT [73] or consequence of TRF2 mutant lacking the basic domain [19], overexpression of the telomerase catalytic subunit [74], or exposure to DNA damaging agents [75].

While typical ALT cell lines display very long telomeres [19], most of CML cells showing ALT characteristics present short telomeres [46,62]. These telomeres of CML samples are similar to those of cells using recombination mechanism in mouse telomerase negative background [76] and normal mammalian somatic cells using ALT [77]. Perhaps, these cells might use the same break induced replication mechanism to maintain their telomere lengths as seen in yeast type I survivors [78]. Thus, it is likely that CML cells during the CP might rely on a similar recombination mechanism as described in these different models to maintain their telomere lengths.

The presence of different ALT phenotypes in CML cells [46,55,62] is highly suggestive of the use of various recombination mechanisms, as it has been proposed for normal and malignant cells [19,23,76,77]. Epigenetic modifications might be a key player governing at least one of these recombination mechanisms. Studies have shown that loss of DNA methylation and heterochromatin marks at subtelomeric regions lead to aberrant telomere elongation through high frequency of telomere recombination [79,80,81,82]. In CML, these epigenetic changes at the telomeric and subtelomeric regions could favor recombination of specific individual telomeres and lead to lengthening of specific individual telomeres, as it has been shown in some CML samples [46,55]. Future studies should look at how subtelomeric and telomeric epigenetic modifications promote the lengthening of some individual telomeres in CML cells and how these epigenetic changes lead to selective lengthening of individual telomeres.

The transition from ALT to telomerase based maintenance mechanism in CML cells may be driven by clonal selection and an increased number of blasts. A correlation between telomerase activity and blast number has been shown as exemplified by a 50-fold increase in telomerase activity in more than 50% of the BC patients [66,83]. It is not known whether these blasts expressing high telomerase activity can maintain residual ALT activity, or a very small fraction of cells at advanced stage of CML can still use solely ALT mechanism. The simultaneous presence of ALT and telomerase in the same cell cannot be ruled out although experimental evidence is lacking to prove this hypothesis in CML. Nevertheless, *in vitro* studies have shown that ALT cells can retain some of their characteristics upon ectopic over expression of telomerase, suggesting that both mechanisms can be present in the same cell [84]. A recent study has weighted in this hypothesis by showing, in human sarcoma, the simultaneous presence of a feature of ALT (APB) and high expression of TERT which correlated with telomerase activity. The same study also found that populations of cells could distinctly use either telomerase or ALT mechanism to maintain their telomeres [85].

**Figure 1:**
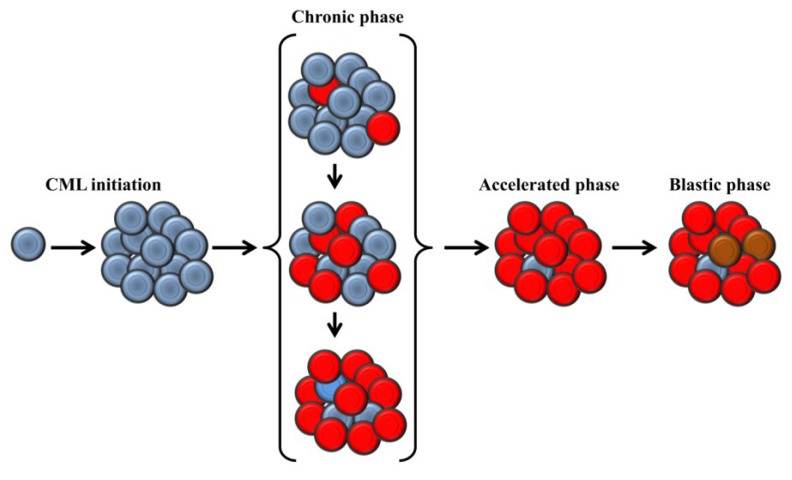
A Suggested model for telomere length maintenance during a course of CML progression. At the initiation of CML, leukemic cells use exclusively alternative lengthening of telomere (ALT) to maintain their telomeres. As CML progresses from an early to a late stage of the chronic phase, clones expressing telomerase become predominant. At the accelerated and blastic phases, the quasi totality of the cells expresses telomerase, but few ALT positive clones remain. At the blastic phase, clones expressing high activity of telomerase appear and become resistant to imatinib. Blue circles: Cells using ALT; Red circles: cells using telomerase; Brown circles: Cells expressing high telomerase activity and resistant to imatinib.

In light of these data, we can infer the possibility that CML cells at an early onset of leukemogenesis might use ALT mechanism only [62]. As the disease advances toward late CP, cells may use both mechanisms; telomerase may become the predominant mechanism as CML evolves from CP to BP. However, persistence of some clones using ALT exclusively or both telomerase and ALT in advanced stage of the disease cannot be ruled out (Figure 1). Some studies are needed to explore mechanisms for telomere length maintenance in different stages of CML to refine the proposed model. Nonetheless, this model can be applied to other hematological malignancies, and a systematic search for ALT markers and telomerase expression level in tumors at different stages of their development can ensure greater effectiveness of any therapy targeting mechanisms maintaining telomere lengths.

## 5. Telomeric Nuclear Organization

The alteration of telomeric nuclear organization has been reported in many cancers [28,31,32,86,87]. However, only two studies have explored telomeric nuclear organization in CML. The first study was done on twelve patients at their diagnosis (CP) and revealed an alteration of telomeric nuclear organization, marked by high number of telomeric aggregates (TAs) as well as changes in telomeric position [87]. This study clearly indicated that an alteration of telomeric nuclear organization could occur at an early clinical phase of a malignancy, as previously hypothesized [35]. Most notably, in this study, patients were categorized into two groups according to the number of TAs: patients with normal number of TAs and patients presenting four times normal level of TAs. However, this study failed to evaluate the clinical values of this high number of TAs because of lack of some clinical information [87]. Nevertheless, an increase number of TAs in CP might be a surrogate for therapeutic response, as shown in Hodgkin lymphoma [86] or might present prognostic values, as reported in glioblastoma [31]. In addition, high number of TAs has been associated with genomic instability [29], and their presence at the CP might be a determinant progression factor from the CP to the AP. A recent study, which had followed eighteen CML patients throughout the three clinical phases by comparing the number of TAs in leukemic cells, has strengthened this hypothesis. This study revealed that the number of TAs increases when patients progress from the CP to the AP and from the AP to the BC [88]. In sum, telomeric nuclear organization might be a predictor for CML progression; most importantly, this malignancy can be used as a model to study mechanisms governing the alteration of telomeric nuclear organization during tumorigenesis.

Besides the presence of telomeric aggregates, it has been shown that telomeres in some CML cells can be either more centrally or peripherally located when they were compared to those of normal cells [87]. Differential positioning of telomeres in CML cells might be a consequence of nuclear remodeling. Human telomeres are attached to nuclear scaffold [89], which is composed of nuclear lamina and intra nuclear scaffold. Loss of Lamina A, a component of the nuclear lamina, led to nuclear peripheral location of telomeres, telomere shortening, loss of telomeric chromatin marks, high frequency of chromosome breakage, and chromosome end to end fusion [90]. Alternatively, change in telomere positioning can lead to nuclear remodeling. Clustering of short telomeres has been involved in the formation of micronuclei, nucleoplasmic bridges, and nuclear buds [91]. Some evidence suggests that telomere positioning is one of the determinant factors governing chromosome territory [29,91,92,93] whose disruption can affect gene expression [94], generate chromosomal abnormalities [29] and disrupt cell function [95]. Moreover, telomeres occupy microterritories throughout the cell cycle, forming groups of telomeres that have peripheral location. Neighboring organization of telomeres favors nuclear organization of chromosomes and their recombination [93,96]. These data raise the possibility that differential positioning of telomeres in CML might be one of the factors involved in gene expression, chromosomal abnormalities, and genomic instability. The advent of high resolution imaging such as the 3D-SIM [97] can help to enhance our understanding on the interplay between the telomeric nuclear organization and nuclear structures during CML evolution.

## 6. Conclusions

Data on telomere lengths, their maintenance mechanisms, and telomeric nuclear organization have provided great insight on the impact of telomere disruption in CML. The concept of telomere shortening, being a feature of CML cells, when average length is only considered has been challenged by the lengthening of some individual telomeres which could possibly be associated with clonal selection. Moreover, the dynamic fluctuation of individual telomere lengths and the presence of *C*-telomeric circles in telomerase negative cells have provided insight on the involvement of ALT for telomere length maintenance in CML cells. ALT might act on the early stage of leukemogenesis, and telomerase might progressively take over telomere length maintenance by jointly carrying out it with ALT or alone. This model might be applied to other hematological malignancies, specifically those of myeloid lineage.

Although efficacious treatments are available for CML patients, new therapeutic resistances are emerging, and anti-telomerase drugs have been a promising therapeutic approach against resistant leukemic cells [69]. However, this anti-telomerase strategy can be hampered by resistant clones which use ALT mechanism. New therapeutic approaches should consider both anti-telomerase and anti-ATL strategies to better target mechanisms of telomere length maintenance in CML and probably in other hematological malignancies.

The nuclear organization of telomeres is likely to predict genomic instability and clinical progression in CML. The emergence of automated and high-resolution microscopies [28,33] would make more efficient and more accurate the study of telomeric nuclear organization and deepen our understanding on the cross talk between telomeres and nuclear structures. Further studies should be undertaking by using CML as a disease model to better understand molecular mechanisms underlying dynamic changes of individual telomere lengths and remodeling of telomeric nuclear organization during oncogenesis.
